# The impact of children’s temperament on recurrent unintentional injuries: the role of paternal parenting styles as a mediator

**DOI:** 10.7717/peerj.14128

**Published:** 2022-10-10

**Authors:** Liuyuan Zhang, Jin Liu, Ying Tang, Li Wang

**Affiliations:** Department of Child and Adolescent Health, Shanxi Medical University, Taiyuan, Shanxi, China

**Keywords:** Children, Children’s temperament, Rejection, Punishing, Recurrent unintentional injuries, Paternal parenting styles

## Abstract

**Background:**

Unintentional injuries (UIs) pose a threat to the health of children all over the world and are a major global health problem. The recurrence of UIs are influenced by the host itself or environmental factors. Children’s temperament and paternal parenting styles (PPS) are important potential factors for poor health outcomes, including recurrent unintentional injuries (recurrent UIs). Therefore, exploring the relationship among these variables may help reduce the likelihood of recurrent UIs in children.

**Purpose:**

To elucidate the mediating effect of PPS in the relationship of children’s temperament characteristics and recurrent UIs among children.

**Methods:**

By multistage random cluster sampling method, a total of 2,850 pupils in grades four and five from 10 different schools were included. The survey included the characteristics of UIs in the past year, parenting styles, and children’s temperament. Structural equation modeling (SEM) was used to examine the possible mediating effect of PPS between children’s temperament and recurrent UIs.

**Results:**

In this study, the incidence rate of recurrent UIs was 3.8%. In temperament, children with recurrent UIs had higher mean scores than the non-recurrent UIs group (*t* = −3.40, −3.52, −3.45, respectively; *p* < 0.001) in the activity, predictability, persistence. Meanwhile, the scores of negative PPS (punishing, over-interference, rejection, and overprotection) were higher in the recurrent UIs group than in the non-recurrent UIs group (*t* = −5.78, −5.05, −5.56, and −3.29; *p* < 0.001, < 0.001, < 0.001, < 0.001, respectively). Using a stepwise binary logistic regression model, grade (OR = 0.23, 95% CI [0.12–0.41], *p* < 0.001), activity (OR = 1.61, 95% CI [1.14–2.26], *p* = 0.007) and over-interference (OR = 2.28, 95% CI [1.37–3.80], *p* = 0.002) had a significant independent relationship with recurrent UIs. The SEM results indicated that children’s temperament was significantly related to negative PPS (*β* = 0.26, *p* < 0.001) and recurrent UIs (*β* = 0.11, *p* = 0.029). The results of the bootstrap test confirmed the significance of the mediating effect of PPS (*β* = 0.06, *p* < 0.001) between children’s temperament and recurrent UIs.

**Conclusions:**

These results suggest that negative PPS plays an important role in mediating children’s temperament and recurrent UIs. It is essential to consider PPS when creating tailored intervention programs to reduce children’s recurrent UIs.

## Introduction

Unintentional injuries (UIs) pose a serious threat to the health of children worldwide and are a major global health concern. The World Health Organization (WHO) reports that more than 900,000 children under the age of 18 die from UIs each year globally (United Nations International Children’s Emergency Fund & World Health Organization, 2008). Meanwhile, UIs may occur repeatedly in children (Braun et al., 2005; Cameron et al., 2016; Junger et al., 2013; Peters, Davies & Van As, 2020); however, they can be prevented and controlled (Tupetz et al., 2020). A previous Australian cohort study of 718 injured children revealed that 29.8% had suffered more than one injury (Cameron et al., 2016). Similarly, a retrospective birth cohort study found that children admitted to the emergency department for two or more UIs accounted for 7.7% (644/8,357) of all children who visited the department (Sever et al., 2019). Recurrent UIs not only affect the children themselves, leading to increased chances of disability and even death, but also adversely affect families and society. Further, patients with a history of injury are 10 times more likely to have another injury than those with no history of injury (Sayfan & Berlin, 1997). Therefore, children with recurrent unintentional injuries (recurrent UIs) are at a high risk for unintentional injury (UI). Identifying high-risk UI groups based on the characteristics of children and their families may allow for refinement in intervention and prevention strategies (Zhang et al., 2016).

There are mainly four behavioral risk factors for UIs in children, namely: demographic risk factors, child-specific risk factors, the influence of parents and other main caregivers, and the role of peers (Schwebel & Gaines, 2007). Demographic characteristics may be a significant factor in unintentional injuries among children. In terms of gender, boys had a significantly higher risk of recurrent UIs than girls (Junger et al., 2013). This may be because boys are physically more active than girls and show more interest in energetic and adventurous activities (Alonso-Fernández et al., 2017). Generally, boys are encouraged to be bold and adventurous, while girls are more likely to be cautioned and viewed as vulnerable (Morrongiello & Lasenby-Lessard, 2007). In addition, the study showed that younger, lower-grade children are at significantly higher risk for recurrent UIs; that is, the age at which children with recurrent UIs suffered their first injury was younger than those of children who had suffered only one injury event (Cameron et al., 2016). Nevertheless, a retrospective cohort study investigated the unintentional injuries of school-age children, and came to the opposite conclusion. They found that the incidence rate of unintentional injuries was higher in high-grade children (Zagel et al., 2019). Furthermore, peer victimization is also a risk factor for unintentional injuries, which is a common factor in the school context of many injured children aged 10–15 years and are mostly unintentional injuries (Laflamme et al., 2003).

Child-specific risk factors include children’s temperament dimensions which are important for children’s health, development, and behavior. Some studies on child characteristics suggest that in children, high hyperactivity scores are positively associated with UI risk (Spinks et al., 2008; Zhang et al., 2016). Cognitive and emotional factors have a strong influence on children’s decision to engage in risky behaviors that threaten their safety (Morrongiello & Matheis, 2004); children with positive or excited emotions around risky activities are more likely to choose risky behaviors, and thus, they are more prone to UIs. These factors are more common in children with difficult temperaments. Children who express difficult temperamental traits may represent more externalizing (*e.g*., hitting or biting) and internalizing (*e.g*., anxiousness or withdrawal) behaviors (Slagt et al., 2016), which are linked to the emergence of unintentional injuries. In summary, children’s temperament characteristics are systematically associated with children’s UIs as they contribute significantly to behavioral risk factors causing UIs.

Although temperament characteristics may predispose a child to behavior trouble, a child who experiences poorer parent parenting style has been linked to increasing risk of injury (Wittig & Rodriguez, 2019). For children, family is one of the most important contexts for their development. Family type has a significant impact on a child’s injury risk. For single parents, the lack of a partner may lead to decreased time and resources for child-rearing. So, it’s more likely to get injuries compared to those from partnered parent families (O’Connor et al., 2000). During the parenting process, suitable behavior (*e.g*., effective communication) may reduce the incidence of injury (Moscato et al., 2021; Wittig & Rodriguez, 2019). Previous research has highlighted the importance of parenting practices in children’s development, and shown that parents play a key role in the occurrence and prevention of injuries. Some types of UIs (such as poisoning, and falls) occur more frequently in younger children than older ones due to parental negligence (Ablewhite et al., 2015; Bhamkar, Seth & Setia, 2016). Permissive parenting has been linked to an increased risk of injury because permissive parents set fewer rules, including rules related to their children’s safety (Schwebel & Gaines, 2007). Additionally, parents who employ a positive parenting style play more with their children which results in more supervision and fewer injuries and thus, lower injury rates (Schwebel et al., 2004). Close supervision and high levels of parental involvement are some dimensions of authoritative parenting. In prior research, children raised in an authoritative parenting style have higher protective and fewer risk behaviors than children from non-authoritative families (Newman et al., 2008). In traditional Chinese culture, parents are stricter and have control over their children (Lai, Zhang & Wang, 2000). The study has shown that the authoritative style, as the advantaged parenting practice, contributes to the physical and mental health of adolescents or young adults (Ding & He, 2022). Hence, a suitable parenting style might prevent young children from experiencing UIs. In most cultures, mothers tend to take the primary responsibility for the child’s growth, but in China, the father holds a more outstanding position of authority in the family, making his involvement in the parenting process more influential on the child’s development (Pan et al., 2016). Pan et al. (2016) have suggested that fathers have a more substantial psychological impact than mothers on children aged 8–12. Most previous studies have focused on the influence of maternal parenting styles on childhood injury, but few have focused on paternal parenting styles (PPS). In fact, PPS is a non-negligible factor in preventing UIs in children. Research illustrates that fathers may play a role in reducing their children’s risk of injury (Fujiwara, Okuyama & Takahashi, 2010). Similarly, some researchers have emphasized the importance of PPS in children’s UI, but its specific role has not been analyzed (Ablewhite et al., 2015; Bhamkar, Seth & Setia, 2016; Schwebel et al., 2004; Schwebel & Gaines, 2007). Nevertheless, the underlying action of PPS between children’s temperament and recurrent UIs remain unclear. Previous studies have linked childhood temperament to UI (Junger et al., 2013), and have suggested that fathers’ parenting styles influence changes in children’s temperament (Möller et al., 2016). These findings imply that PPS may mediate the relationship between children’s temperament and recurrent UIs in children. To date, no research has evaluated the mediating role of PPS in this relationship. This study aimed to elucidate the mediating effect of PPS between children’s temperament and recurrent UIs among children.

Thus, this study aimed to elucidate the relationship among recurrent UIs (defined as three or more UIs occurring in 1 year), host factors (children’s inherent temperament), and environmental factors (PPS) by applying SEM. We hypothesized that, first, there would be different demographic characteristics, temperament characteristics and PPS between children with recurrent UIs and the non-recurrent UIs. Second, there would be a significant positive association between children’s temperament characteristics and recurrent UIs among children. Third, the relationship between children’s temperament and recurrent UIs would be mediated by PPS. This study provides a reference for the development of effective prevention strategies for recurrent UIs in children.

## Methods

### Participants

**Figure 1:**
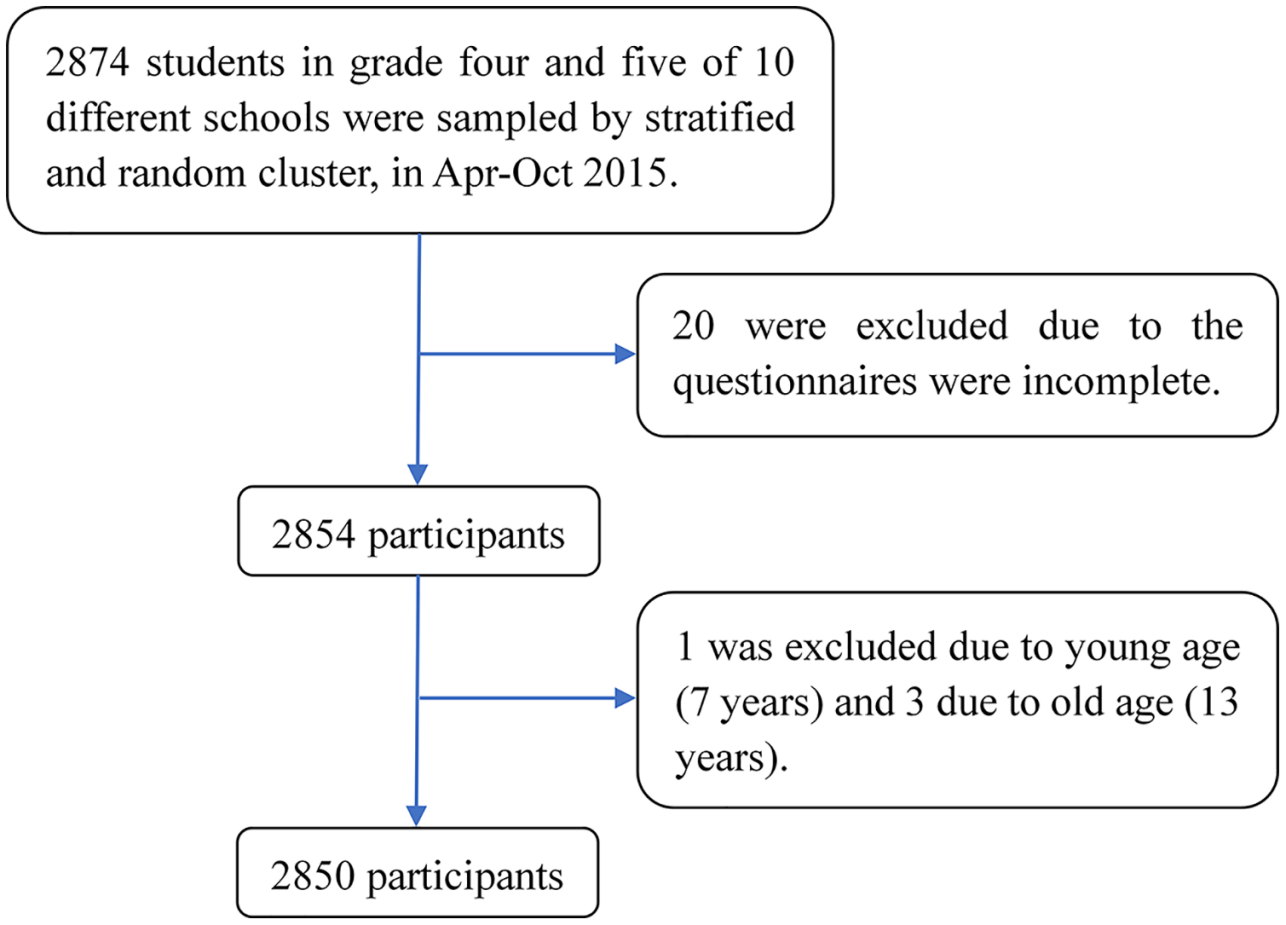
Flowchart of the participants selection.

This was a retrospective cross-sectional survey using a multistage random cluster sampling method to recruit participants. The sample was stratified by area (urban *vs* suburban). Random cluster sampling was then used to choose classes from grades four and five level for each selected school. A total of 2,874 samples were recruited from 10 different schools in Taiyuan, China, from April to October 2015. All 2,874 children participated in the survey and returned questionnaires on time. Of these, 24 questionnaires were excluded because of incomplete data and the age of the child, and 2,850 questionnaires were obtained, leading to a response rate of 99.16%. The final sample of the study comprised 2,850 children (Fig. 1), including 52.2% (*n* = 1,488) male and 47.8% (*n* = 1,362) female, with 53.6% (*n* = 1,529) of cases coming from the fourth grade and 46.4% (*n* = 1,321) from fifth grade. The participants’ ages ranged from 8 to 12 years old (*M* = 9.7, *SD* = 0.8), and 83.7% (*n* = 2,386) of the participants were aged between 9 and 10 years. Their parents or guardian were also invited to participate in the study to assess children’s temperament. Eventually, 2,662 completed MCTQs were collected, with an effective response rate of 92.6%. This study was reviewed and approved by the Shanxi Medical University Ethics Committee (2014021).

### Diagnostic criteria for unintentional injury

Unintentional injury is defined as any damage to bodily tissues incurred through non-intentional (that is, not abusive or purposefully self-inflicted) channels (Schwebel & Barton, 2006). According to the International Classification of Diseases and Related Health Problems, 10th Revision (World Health Organization, 2010), UIs are divided into 10 categories, namely: road traffic injuries, falls, burns/scalds, crushing injuries, animal bites, scratches, poisoning, drowning, suffocation, and other injuries. In this study, these categories and criteria were adopted to measure UIs in the sample. Any one of the following two situations can be considered a UI (Wang, 2005): (1) being diagnosed with a certain kind of unintentional injury after treatment by a medical unit; (2) rest (leave of absence) for one day or more due to unintentional injury.

### Measurements of unintentional injury measures

Children were asked to complete a UI history questionnaire with demographic and background information (grade, gender, date of birth, residence, only child, *etc*.) and UIs (number and type of injury) in the previous 12 months. Before children were asked whether they had suffered UIs in the previous 12 months, we introduced what an UI is. If the child had suffered unintentional injury, he/she will answer the number of times and choose the cause. (Question-1 “Did you suffer unintentional injury in the previous 12 months?” Question-2 “How many unintentional injuries have you had in the previous 12 months?”, Question-3 “Please choose the cause of your unintentional injury in the previous 12 months?”). It should be emphasized that the questionnaire was designed by the researchers.

Subjects with three or more incidents of UIs in the previous 12 months were considered to have recurrent UIs (Banerjee & Yadav, 2021).

### PPS

The Egna Minnen Beträffande Uppfostran (EMBU) questionnaire was developed by Perris in Sweden to assess parents’ parenting styles. Yue et al. (1993) took the English scale as the original scale, translated and revised the EMBU in combination with China’s national conditions, and formed the Chinese version of the Parenting Style Scale, which has been widely used in China. In this study, the revised Chinese version of the EMBU scale was used. It consists of a previously validated 58-item scale and includes six dimensions of PPS (emotional warmth, punishing, over-interference, favoritism, rejection and overprotection) (Table 1). Across all EMBU dimensions, a higher score can be interpreted as a higher frequency with which the parent engaged in a particular parenting style dimension. High scores for parental rejection, punishing, over-interference, over-protection, and favoritism and low scores on emotional warmth were deemed as negative parenting styles. In this study, the Chinese version of the EMBU used in this study is valid and reliable (Yue et al., 1993). The internal consistency (0.50–0.89) and retest reliability (0.63–0.73) of the six dimensions of paternal rearing styles were satisfactory.

**Table 1:** Subscales and sample items of PPS in EMBU.

Subscale	Sample items	Number of items
Emotional warmth	I felt that my parents liked me.	19
Punishing	My parents punished me for even small offences.	12
Over-interference	My parents wanted to decide how I should be dressed or how I should look.	10
Favoritism	I felt that my parents liked me more than they liked my sister(s) and/or brother(s).	5
Rejection	My parents said that they did not approve of my behaviour at home.	6
Overprotection	My parents always worried too much about my health.	6

### Children’s temperament

Children’s temperament was evaluated using the Middle Childhood Temperament Questionnaire (MCTQ). The MCTQ is a parental report questionnaire designed to measure children’s behavior on a six-point Likert scale ranging from “almost never” to “almost always”. The scale used in this study is the Chinese version of the temperament Scale translated and revised by Zhang, Xu & Shen (2000) on the basis of the original MCTQ scale. All the translations and adaptations of the Chinese version are authorized and approved by the original author (Zhang, Xu & Shen, 2000). It consists of 99 items and assesses the following nine temperament categories: activity, predictability, approach, adaptability, intensity, mood, persistence, distractibility, and threshold. The internal consistency and retest reliability of nine subscales were (0.76–0.86) and (0.80–0.93), respectively (Zhang, Xu & Shen, 2000). The scores of each dimension of the scale were calculated by temperament evaluation software for aged 8–12 children (Children’s Health Department and Information Center of Xinhua Hospital Affiliated to Shanghai Second Medical University).

### Procedure

In the classroom, trained investigators explained the purpose of the survey to the students. The students completed the UI and EMBU self-report questionnaires in 20 min. The MCTQ and the informed consent were delivered by student to his/her parents or guardian. The parents or guardian signed the informed consent, completed the MCTQ within two working days, and return it to the school through their children. Subsequently, the investigators checked the accuracy and completeness of the returned questionnaires to exclude questionnaires that did not qualify.

### Statistical analysis

After unified coding of the eligible questionnaires, Epidata 3.1 software was used to construct the database. Questionnaire data entry was conducted, and double-entry verification was applied to ensure the data was entered correctly. Descriptive analysis of qualitative data (grade, gender, occurrence of recurrent UIs, *etc*.) was conducted using frequencies and percentages. Chi-square tests were used to compare the frequencies of recurrent UIs on different levels of categorical socio-demographic factors. To analyze the associations between recurrent UIs and age, a p-value for the trend test (*χ*^*2*^_trend_) was calculated. Quantitative variables, such as the MCTQ and EMBU scores, were expressed as the mean ± standard deviation (SD), and t-tests were used to compare the score differences between groups (recurrent UIs group and non-recurrent UIs group). Forward stepwise binary logistic regression analysis was used to determine the influencing factors of recurrent UIs. All analyses were performed using Statistical Package for the Social Sciences (SPSS), version 22.0 (SPSS, Inc., Chicago, IL, USA). Significance was assessed using a two-tailed test, with a critical value of 0.05. We also performed Pearson’s correlation coefficients to describe the relationship between MCTQ and EMBU (Table S1). A heat map of the Pearson product correlation coefficients between MCTQ and EMBU (Fig. S1) was drawn using Origin version 2022 software (Origin Lab, Northampton, MA, USA).

Confirmatory factor analyses (CFA) on continuous variables were conducted using Mplus version 6.11 (Muthén & Muthén, Los Angels, CA, USA) to construct each known latent variable. Models were considered as well-fitting if the root mean square error of approximation (RMSEA) was <0.08, comparative fit index (CFI) was >0.95, Tucker-Lewis index (TLI) was >0.95, and standardized root mean square residual (SRMR) was = 0.08 (Hu & Bentler, 1998; Wang et al., 2018).

Structural equation modeling (SEM) was used as a confirmatory technique to test the hypothesized mediation model. SEM allows simultaneous evaluation of the direct, indirect, and total effects of a set of predictor variables on multiple mediators and outcome variables. SEM also allows the construction of latent variables that are not measured directly, but are estimated from measured variables. Parameter estimations for the SEM models were conducted using the weighted least squares with mean and variance (WLSMV) adjusted for the *χ*^*2*^ method, which was suitable for dealing with categorical dependent variables. Modifications were made to the hypothetical model to achieve a better-fitting and parsimonious model. Models were considered well-fitting if the weighted root mean residual (WRMR) was <0.90 (Wang et al., 2018). A bootstrap test was used to test the mediating effect. Bootstraps with 1,000 resamples were applied to these analyses. Note that we use the term “mediate” only in the statistical sense, as it is difficult to ascertain the causal direction of an association using cross-sectional data. The statistical significance for standardized estimates (*β*) from the SEM was set at *p* < 0.05.

## Results

### Characteristics of UI

The proportion of children who experienced UI was 23.1% (*n* = 658). Additionally, 404 children were injured once, 145 children were injured twice, and 109 children were injured three or more times. The prevalence rate of recurrent UIs was 3.8% (109/2,850). We investigated the occurrence of UI within one year. During this period, 916 injuries were reported. Therefore, the overall incidence of UI among 8–12 years old children in this study was 32.7 injury events per 100 children during the study period. Some participants could not remember the specific types of UI they experienced. The most frequently reported type of UI was falls (31.9%, 292/916), followed by scratches (21.4%, 196/916), and animal bites (14.3%, 131/916).

### Demographic characteristics of recurrent UIs

The distribution of recurrent UIs in children was significantly related to gender and age. The proportion of recurrent UIs in males (5.4%) was higher than that in females (2.1%), and the proportion of recurrent UIs in the fourth grade (5.0%) was twice that of the fifth grade (2.5%). These were statistically significant (*p* < 0.001, < 0.001, respectively). Further analysis using linear-by-linear association indicated that recurrent UIs were correlated with age, suggesting that with an increase in age, the proportion of recurrent UIs declined (*χ*^*2*^_*trend*_ = 4.27, *p* = 0.039) (Table 2). Therefore, boys and younger children are more likely to have recurrent unintentional injuries.

**Table 2:** General characteristics and recurrent unintentional injuries among in children.

Variable	*N* (%)	Recurrent UIs *n* (%)	Non-recurrent UIs *n* (%)	*χ* ^*2*^	*p*
Gender				22.188	<0.001***
Male	1,488 (52.2)	81 (5.4)	1,407 (94.6)		
Female	1,362 (47.8)	28 (2.1)	1,334 (97.9)		
Grade				11.778	<0.001***
4	1,529 (53.6)	76 (5.0)	1,453 (95.0)		
5	1,321 (46.4)	33 (2.5)	1,288 (97.5)		
Age^a^				4.269	0.039*
8–9	1,266 (44.4)	61 (4.8)	1,205 (95.2)		
10	1,219 (42.8)	36 (3.0)	1,183 (97.0)		
11–12	365 (12.8)	12 (3.3)	353 (96.7)		
Residence				1.190	0.275
Urban	1,607 (56.4)	67 (4.2)	1,540 (95.8)		
Rural	1,243 (43.6)	42 (3.4)	1,201 (96.6)		
Only child				0.566	0.452
Yes	958 (33.6)	33 (3.4)	925 (96.6)		
No	1,892 (66.4)	76 (4.0)	1,816 (96.0)		
Mother is alive^b^				–	0.349
Yes	2,838 (99.6)	108 (3.8)	2,730 (96.2)		
No	11 (0.4)	1 (9.1)	10 (90.9)		
Missing	1 (0.0)				
Father is alive^c^				0.000	1.000
Yes	2,822 (99.0)	108 (3.8)	2,714 (96.2)		
No	27 (0.9)	1 (3.7)	26 (96.3)		
Missing	1 (0.1)				
Parents divorced^c^				0.003	0.956
Yes	94 (3.3)	3 (3.2)	91 (96.8)		
No	2,752 (96.6)	106 (3.9)	2,646 (96.1)		
Missing	4 (0.1)				

**Notes:**

UIs, unintentional injuries.

a linear-by-linear association.

b Fisher’s exact test.

c continuity correction.

* *p* < 0.05.

*** *p* < 0.001.

### Distribution of recurrent UIs in different child temperament characteristics and PPS

As Table 3 shows, the mean scores of the three temperamental characteristics (activity, predictability, and persistence) in the recurrent UIs group were higher than those in the non-recurrent UIs group (*t* = −3.40, −3.52, and −3.45, respectively; *p* < 0.001). Moreover, we discovered that children with recurrent UIs obtained higher mean scores on negative PPS dimensions such as punishing, over-interference, rejection, and overprotection (*t* = −5.78, −5.05, −5.56, and −3.29; *p* < 0.001, < 0.001, < 0.001, < 0.001, respectively). Using a stepwise binary logistic regression model, grade, activity and over-interference had a significant independent relationship with recurrent UIs. Results showed that, when compared with pupils in grade four, pupils in grade five (OR = 0.23, 95% CI [0.12–0.41], *p* < 0.001) appeared a protective factor for recurrent UIs. Moreover, pupils who scored higher on dimensions of “activity” (OR = 1.61, 95% CI [1.14–2.26], *p* = 0.007) and “over-interference” (OR = 2.28, 95% CI [1.37–3.80], *p* = 0.002) showed higher risk in recurrent UIs. Pearson product correlations and *p* values were also calculated to assess the relationship between the degree of the nine temperament categories in MCTQ and the scores of the six dimensions of PPS in EMBU (Fig. S1 and Table S1). There were some weak but statistically significant correlations (*e.g*., *r* = −0.18 (emotional warmth and mood), 0.180 (punishing and persistence), and 0.155 (rejection and mood); *p* < 0.001).

**Table 3:** Comparison of the mean (SD) scores of temperamental characteristics and paternal parenting styles between recurrent unintentional injuries children and children not with unintentional injuries.

Subscale	Recurrent UIs (*n* = 109)	Non-recurrent UIs (*n* = 2,741)	*t*	*p*
MCTQ				
Activity	3.14 (0.73)	2.89 (0.72)	−3.399	<0.001***
Predictability	3.21 (0.48)	3.04 (0.57)	−3.515	<0.001***
Approach	3.21 (0.66)	3.31 (0.64)	1.602	0.109
Adaptability	2.92 (0.65)	2.85 (0.66)	−1.078	0.281
Intensity	3.22 (0.79)	3.08 (0.78)	−1.803	0.071
Mood	2.97 (0.59)	2.85 (0.61)	−1.870	0.062
Persistence	2.98 (0.76)	2.71 (0.75)	−3.453	<0.001***
Distractibility	3.95 (0.57)	3.97 (0.70)	0.277	0.782
Threshold	3.52 (0.68)	3.54 (0.75)	0.176	0.861
EMBU				
Emotional warmth	2.69 (0.59)	2.70 (0.54)	0.194	0.847
Punishing	1.89 (0.63)	1.55 (0.54)	−5.782	<0.001***
Over-interference	2.26 (0.52)	1.98 (0.43)	−5.045	<0.001***
Favoritism	2.14 (0.68)	2.06 (0.64)	−0.921	0.357
Rejection	1.85 (0.61)	1.56 (0.52)	−5.564	<0.001***
Overprotection	2.38 (0.61)	2.19 (0.54)	−3.290	<0.001***

**Notes:**

UIs, unintentional injuries.

*** *p*< 0.001.

### Mediation analyses

**Figure 2:**
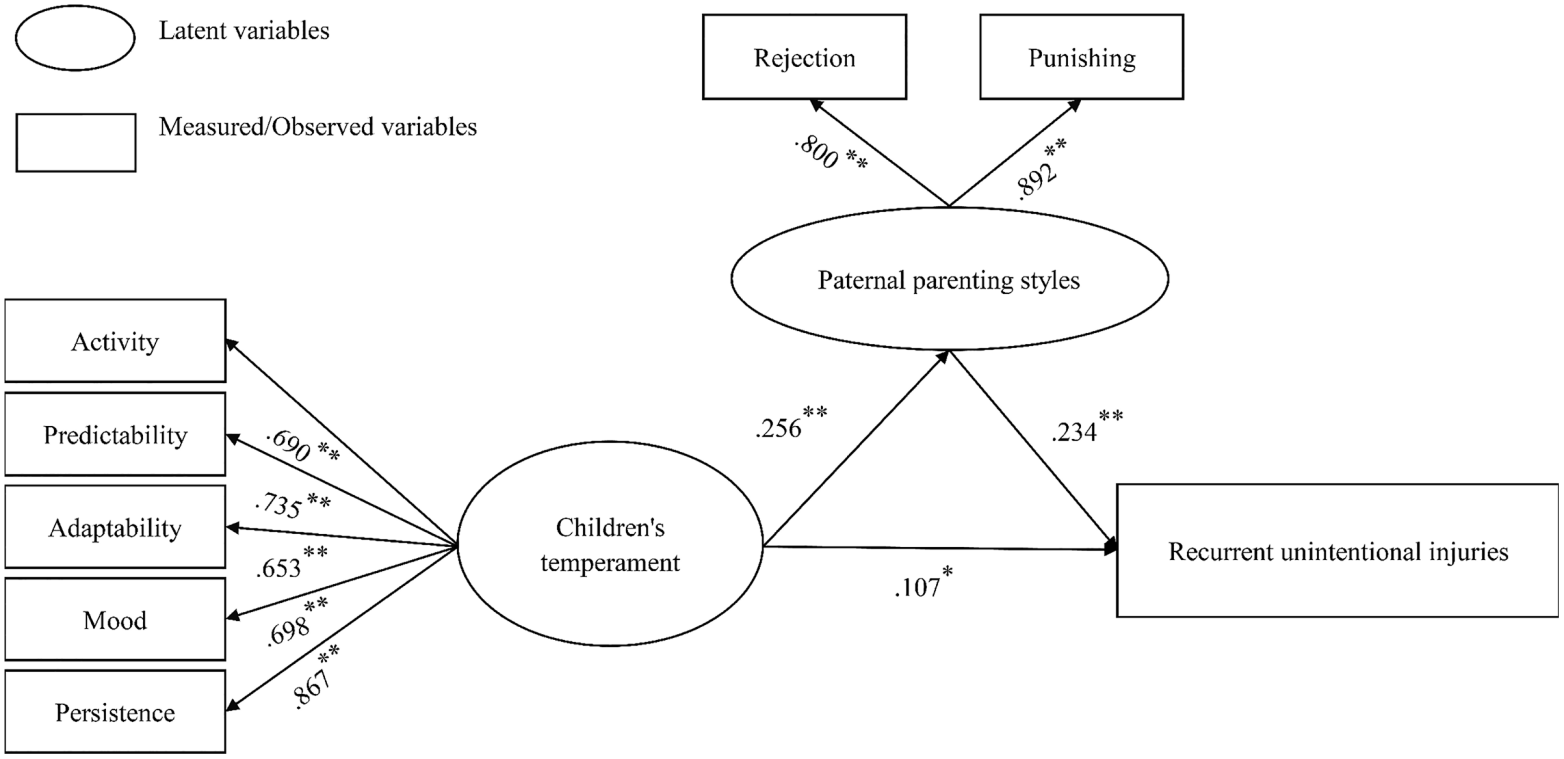
The mediation model involving negative paternal parenting styles mediates the relation between children’s temperament and recurrent unintentional injuries. *p < 0.05, **p < 0.01

Confirmatory factor analysis was used for these two potential variables, that is, five temperamental characteristics measuring children’s temperament and two paternal factors measuring negative PPS fit well into the confirmatory factor analysis model (RMSEA = 0.04, CFI = 0.99, TLI = 0.99, SRMR = 0.02). The SEM results showed that: first, children’s temperament was significantly related to recurrent UIs and negative PPS; second, negative PPS was significantly related to recurrent UIs; third, the impact of children’s temperament on recurrent UIs reduced after negative PPS was controlled. The SEM of the hypothetical mediation model for recurrent UIs resulted in a good fit (WRMR < 0.66). The results of the bootstrap test confirmed the significance of the mediating effect of PPS (*β* = 0.06, *p* < 0.001). As Fig. 2 shows, child temperament had a direct effect on recurrent UIs (*β* = 0.11, *p* = 0.03) and negative PPS (*β* = 0.26, *p* < 0.001). The pathway from PPS to recurrent UIs was also significant (*β* = 0.23, *p* < 0.001), with high levels of negative PPS predicting more recurrent UIs. Thus, the results supported the second hypothesis.

## Discussion

The present study examined the relationship between PPS, child temperament, and recurrent UIs in grade four and five children (8–12 years old) using data reported by children and their parents. Recurrent UIs were reported retrospectively by the study participants. The children’s questionnaire lists UI cause categories and the number of UIs a child has suffered in a year. In this study, the frequency of three or more accidents is defined as “recurrent UIs”, which can be used to screen children at higher risk of injury. The results of the present study suggest that the prevalence rate of recurrent UIs is 3.8%. Recurrent UIs occurred among a minority of children. This is consistent with previous studies (Banerjee & Yadav, 2021; Junger et al., 2013). Consistent with most studies, after adjusting for covariates, we found that higher grade (grade five) was a protective factor, indicating that higher grade was associated with lower risk of recurrent UIs. We also found that the activity of MCTQ and the over-interference of EMBU were risk factors for recurrent UIs; that is, the higher the score of “activity” or “over-interference”, the greater the possibility of recurrent UIs. These results are consistent with Schwebel’s study that greater activity level predicts higher injury rates (Schwebel et al., 2011). When children with high activity level participate in activities, they easily make judgments without thinking, overestimate their physical fitness, and lead to injury (Schwebel, 2003). In addition, a highly supervised pattern of behavior is adopted by “helicopter-parents” (*i.e*., over-interference parents) for their child (Doepke & Zilibotti, 2019). However, over-interference, which is equivalent to pushing children into the dilemma of negative growth, is easy to make children have low self-esteem, sensitivity, anxiety, and other negative emotions (Baginsky & Manthorpe, 2021). These helicopter-parents practices increase the risk of injuries to their children. Another possible reason is that Chinese fathers are more concerned about their children’s studies (Huang et al., 2015), and less care about their children’s daily activities. So, even with their over-intervene, the child is still prone to unintentional injuries.

Under the influence of their original family and the collection of their own experiences, each parent forms their own distinctive parenting style. Correspondingly, their children have their own characteristics, which leads to them forming different temperaments. Children’s temperament also has an impact on parenting styles. For example, children with poor adaptability and negative attitudes towards trouble may have negative parenting styles (punishment, abuse, *etc*.). Previous studies have also shown that children’s temperament and parenting style can affect the occurrence of UI (Cameron et al., 2016; Schwebel & Gaines, 2007). Although child temperament may lead to different internalized or externalized behaviors, the developmental results of children are also affected by their parents. In other words, parents are an important social force in children’s development (Wittig & Rodriguez, 2019). However, few studies have focused on the role of PPS in children’s temperament and experience of UI. In line with earlier findings, the results of this study showed a relationship between children’s temperament and recurrent UIs. Nevertheless, our study find that negative PPS plays an important role in mediating children’s temperament characteristics and recurrent UIs. No similar results are found in literatures. This suggests that higher recurrent UI rates generally reported in children with higher activity, predictability, and persistence scores may be due to the fact that these children’s fathers present an increased level of negative PPS. Fathers who adopt a negative parenting style may create an environment that encourages children to act out and take risks. Therefore, these children may be at a higher risk of experiencing recurrent UIs. In addition, the temperament characteristics of children with a higher risk of UI have been discussed not to blame the victim, but to better identify children who are at risk, factoring the psychological, family, and social aspects, to better protect high-risk children against UI.

This study has some limitations. First, because the experience of UI was questioned only once in this survey, the data were cross-sectional, limiting the causal interpretation that we could draw from the results. Meantime, in this study, the number of unintentional injuries was mainly analyzed, and the cause of injuries were not analyzed, which will be explored in the future study. Second, children’s self-reporting of unintentional injuries in the previous 12 months may be subject to recall bias. Third, it is important to note that mothers’ and fathers’ parenting styles are interdependent and may influence each other. Future research should use longitudinal designs to test the proposed mediation model in a more robust manner.

The strengths of this study help to extend research on unintentional injuries and child temperament in several ways. Our study highlights the crucial mediating role of paternal parenting between children’s UIs and children’s temperament, implying that father’s poor parenting style plays a non-negligible role in children’s UIs. In addition, we explored the effects of children’s sex, age, and subscale on the recurrence of unintentional injuries and found some interesting results. Another strength of our study is that the sample size was relatively large, which increases the practical significance, and the conclusions may be more applicable to China.

This study combined children’s temperament (as the host factor) and PPS (as an environmental factor) to explore the causes of recurrent UIs. The results showed that negative PPS played a mediating role in the relationship between child temperament and recurrent UIs in children. Therefore, in studies on UI prevention among children, both host factors and environmental factors should be concerned at the same time. Fathers should be involved in the prevention of UIs in children and adopt positive parenting style according to the characteristics of the children’s temperament to reduce the occurrence of repetitive UIs.

## Conclusions

This study provides novel insight into the association between children’s temperament and recurrent UIs, highlighting the mediating role of PPS. Results intimate that negative PPS plays an important role in mediating children’s temperament and recurrent UIs, *i.e*., high levels of negative PPS predicts more recurrent UIs. These findings may inform the development of targeted public health interventions, and future prevention and intervention strategies could be targeted to fathers to prevent recurrent UIs among children.

## Supplemental Information

10.7717/peerj.14128/supp-1 Supplemental Information 1 Heat map of Pearson’s correlation coefficients between the degree of the nine temperament categories in MCTQ and the scores of the six dimensions of PPS in EMBU. Heat map of Pearson’s correlation coefficients between the degree of the nine temperament categories in MCTQ and the scores of the six dimensions of PPS in EMBU.

10.7717/peerj.14128/supp-2 Supplemental Information 2 Comparison of Pearson’s correlation coefficients between the degree of the nine temperament categories in MCTQ and the scores of the six dimensions of PPS in EMBU. *p < 0.05, **p < 0.01, ***p < 0.001.

10.7717/peerj.14128/supp-3 Supplemental Information 3 Raw data including general information of the subjects and scale scores.

10.7717/peerj.14128/supp-4 Supplemental Information 4 Statistical reporting.

## Additional Information and Declarations

### Data Availability

The following information was supplied regarding data availability: The raw data and statistical reports are available in the Supplemental Files.
