# Supplementary figures and images for: The impact of children’s temperament on recurrent unintentional injuries: the role of paternal parenting styles as a mediator
Source: PeerJ. 2022 Oct 10;10:e14128. doi: 10.7717/peerj.14128 (PMC9559059; doi:10.7717/peerj.14128)

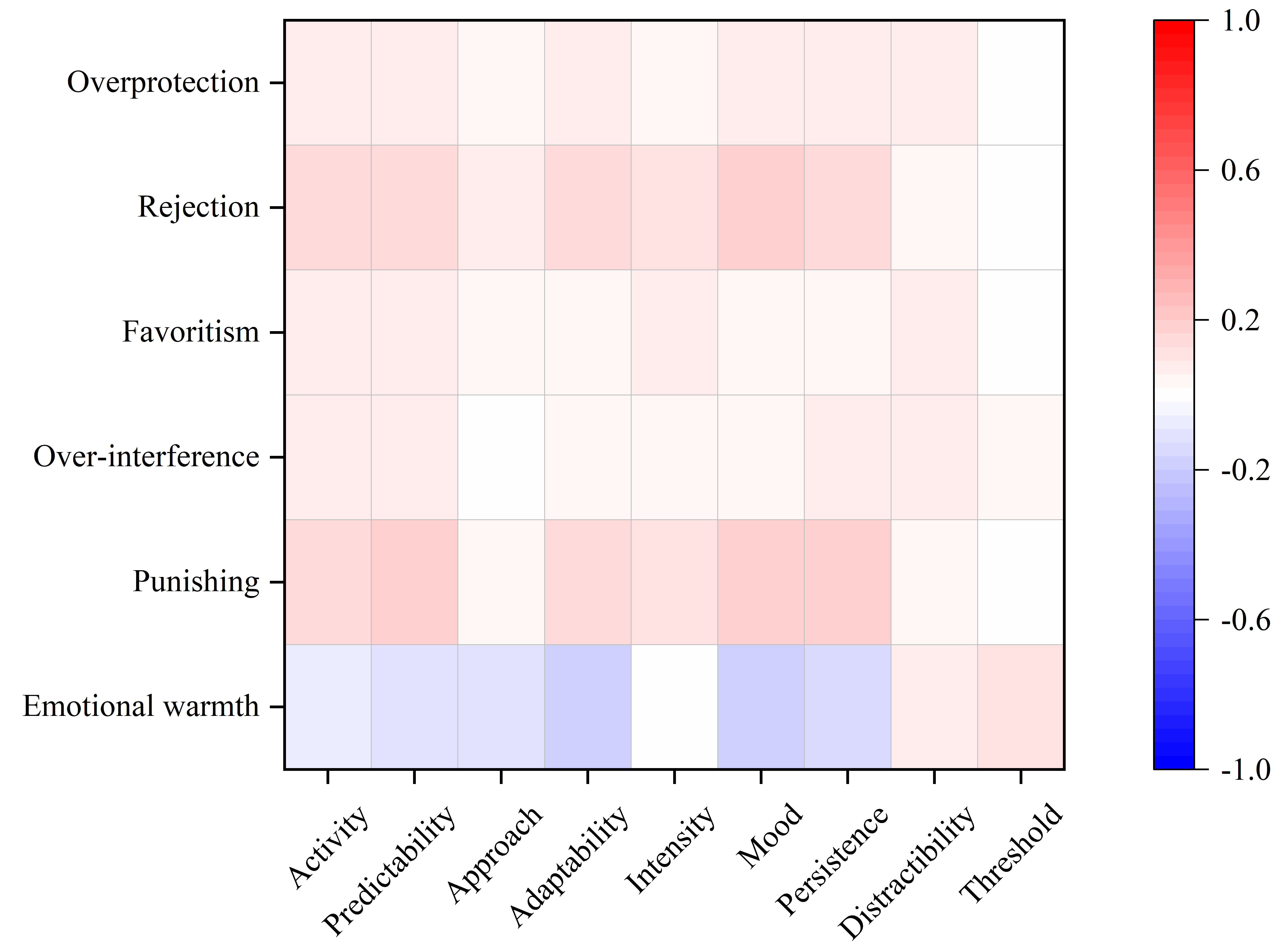

Supplement: Supplemental Information 1 — Heat map of Pearson’s correlation coefficients between the degree of the nine temperament categories in MCTQ and the scores of the six dimensions of PPS in EMBU. [file peerj-10-14128-s001.png]
