# Supplementary material for: The impact of children’s temperament on recurrent unintentional injuries: the role of paternal parenting styles as a mediator
Source: PeerJ. 2022 Oct 10;10:e14128. doi: 10.7717/peerj.14128 (PMC9559059; doi:10.7717/peerj.14128)
Supplement: Supplemental Information 2 — *p < 0.05, **p < 0.01, ***p < 0.001. [file peerj-10-14128-s002.docx]

|  | Activity | Predictability | Approach | Adaptability | Intensity | Mood | Persistence | Distractibility | Threshold |
| --- | --- | --- | --- | --- | --- | --- | --- | --- | --- |
| Emotional warmth | -0.063^**^ | -0.101^***^ | -0.120^***^ | -0.175^***^ | -0.011 | -0.177^***^ | -0.146^***^ | 0.055^**^ | 0.113^***^ |
| Punishing | 0.153^***^ | 0.168^***^ | 0.046^*^ | 0.163^***^ | 0.099^***^ | 0.177^***^ | 0.180^***^ | 0.041^*^ | -0.009 |
| Over-interference | 0.076^***^ | 0.072^***^ | -0.006 | 0.052^*^ | 0.046^*^ | 0.038 | 0.056^**^ | 0.083^***^ | 0.025 |
| Favoritism | 0.057^*^ | 0.084^**^ | 0.026 | 0.040 | 0.065^**^ | 0.040 | 0.045 | 0.058^*^ | 0.018 |
| Rejection | 0.142^***^ | 0.147^***^ | 0.062^**^ | 0.147^***^ | 0.093^***^ | 0.165^***^ | 0.160^***^ | 0.025 | 0.001 |
| Overprotection | 0.073^***^ | 0.088^***^ | 0.041^*^ | 0.087^***^ | 0.050^*^ | 0.088^***^ | 0.078^***^ | 0.058^**^ | 0.011 |
