# Supplementary material for: The impact of children’s temperament on recurrent unintentional injuries: the role of paternal parenting styles as a mediator
Source: PeerJ. 2022 Oct 10;10:e14128. doi: 10.7717/peerj.14128 (PMC9559059; doi:10.7717/peerj.14128)
Supplement: Supplemental Information 4 [file peerj-10-14128-s004.docx]

We use chi-square test to check the comparability of two sets of counting data.

| Variable | Recurrent UIs  N (%) | Non-recurrent UIs  N (%) | *χ^2^* | *P* | *v* | η |
| --- | --- | --- | --- | --- | --- | --- |
| Gender |  |  | 22.188 | <0.001 | 1 | 0.088 |
| Male | 81(5.4) | 1407(94.6) |  |  |  |  |
| Female | 28(2.1) | 1334(97.9) |  |  |  |  |
| Grade |  |  | 11.778 | 0.001 | 1 | 0.064 |
| 4 | 76(5.0) | 1453(95.0) |  |  |  |  |
| 5 | 33(2.5) | 1288(97.5) |  |  |  |  |
| Age^a^ |  |  | 4.269 | 0.039 | 2 | 0.070 |
| 8-9 | 61(4.8) | 1205(95.2) |  |  |  |  |
| 10 | 36(3.0) | 1183(97.0) |  |  |  |  |
| 11-12 | 12(3.3) | 353(96.7) |  |  |  |  |
| Residence |  |  | 1.190 | 0.275 | 1 | 0.200 |
| Urban | 67(4.2) | 1540(95.8) |  |  |  |  |
| Rural | 42(3.4) | 1201(96.6) |  |  |  |  |
| Only child |  |  | 0.566 | 0.452 | 1 | 0.014 |
| Yes | 33(3.4) | 925(96.6) |  |  |  |  |
| No | 76(4.0) | 1816(96.0) |  |  |  |  |
| Mother is alive^b^ |  |  | – | 0.349 | 1 | 0.017 |
| Yes | 108(3.8) | 2730(96.2) |  |  |  |  |
| No | 1(9.1) | 10(90.9) |  |  |  |  |
| Missing |  |  |  |  |  |  |
| Father is alive^c^ |  |  | 0.000 | 1.000 | 1 | 0.000 |
| Yes | 108(3.8) | 2714(96.2) |  |  |  |  |
| No | 1(3.7) | 26(96.3) |  |  |  |  |
| Missing |  |  |  |  |  |  |
| Parents divorced^c^ |  |  | 0.003 | 0.956 | 1 | 0.006 |
| Yes | 3(3.2) | 91(96.8) |  |  |  |  |
| No | 106(3.9) | 2646(96.1) |  |  |  |  |
| Missing |  |  |  |  |  |  |

UIs=unintentional injuries; ^a^linear-by-linear association; ^b^Fisher's exact test; ^c^continuity correction

When the skewness and kurtosis are ≈ 0, the distribution can be considered to be symmetric and follow normal distribution. When the kurtosis is ≈0, the distribution can be considered to have a suitable peak and follow a normal distribution. When the sample size is large, the sample can also obey the normal distribution by default.

|  | skewness | Standard errors of skewness | kurtosis | Standard errors of kurtosis |
| --- | --- | --- | --- | --- |
| MCTQ |  |  |  |  |
| Activity | 0.046 | 0.047 | -0.279 | 0.095 |
| Predictability | -0.058 | 0.047 | -0.152 | 0.095 |
| Approach | -0.07 | 0.047 | 0.227 | 0.095 |
| Adaptability | -0.166 | 0.047 | -0.172 | 0.095 |
| Intensity | 0.012 | 0.047 | -0.445 | 0.095 |
| Mood | -0.103 | 0.047 | -0.241 | 0.095 |
| Persistence | 0.085 | 0.047 | -0.335 | 0.095 |
| Distractibility | -0.197 | 0.047 | -0.003 | 0.095 |
| Threshold | -0.196 | 0.047 | 0.070 | 0.095 |
| EMBU |  |  |  |  |
| Emotional warmth | -0.279 | 0.050 | -0.372 | 0.100 |
| Punishing | 1.537 | 0.049 | 2.466 | 0.098 |
| Over-interference | 0.572 | 0.048 | 0.103 | 0.097 |
| Favoritism | 0.343 | 0.059 | -0.452 | 0.117 |
| Rejection | 1.326 | 0.047 | 1.993 | 0.094 |
| Overprotection | 0.305 | 0.048 | -0.219 | 0.095 |

Homogeneity of variance test.

|  | *V_1_* | *V_2_* | *P* |
| --- | --- | --- | --- |
| MCTQ |  |  |  |
| Activity | 1 | 1154 | 0.612 |
| Predictability | 1 | 1154 | 0.023 |
| Approach | 1 | 1154 | 0.871 |
| Adaptability | 1 | 1154 | 0.472 |
| Intensity | 1 | 1154 | 0.769 |
| Mood | 1 | 1154 | 0.107 |
| Persistence | 1 | 1154 | 0.412 |
| Distractibility | 1 | 1154 | 0.345 |
| Threshold | 1 | 1154 | 0.745 |
| EMBU |  |  |  |
| Emotional warmth | 1 | 1154 | 0.008 |
| Punishing | 1 | 1154 | 0.079 |
| Over-interference | 1 | 1154 | 0.003 |
| Favoritism | 1 | 1154 | 0.293 |
| Rejection | 1 | 1154 | 0.330 |
| Overprotection | 1 | 1154 | 0.521 |

Comparison between groups was performed using the T-test.

|  | Recurrent UIs  (N=109) | Non-recurrent UIs  (N=2741) | *T* | *P* | *V* | *95%CI* | *Cohen's d* |
| --- | --- | --- | --- | --- | --- | --- | --- |
| MCTQ |  |  |  |  |  |  |  |
| Activity | 3.14(0.73) | 2.89(0.72) | -3.399 | 0.001 | 2659 | (0.105,0.393) | 0.35 |
| Predictability | 3.21(0.48) | 3.04(0.57) | -3.515 | 0.001 | 111.54 | (0.075,0.267) | 0.32 |
| Approach | 3.21(0.66) | 3.31(0.64) | 1.602 | 0.109 | 2659 | (-0.233,0.023) | 0.15 |
| Adaptability | 2.92(0.65) | 2.85(0.66) | -1.078 | 0.281 | 2659 | (-0.059,0.202) | 0.11 |
| Intensity | 3.22(0.79) | 3.08(0.78) | -1.803 | 0.071 | 2659 | (-0.013,0.299) | 0.18 |
| Mood | 2.97(0.59) | 2.85(0.61) | -1.870 | 0.062 | 2659 | (-0.006,0.236) | 0.20 |
| Persistence | 2.98(0.76) | 2.71(0.75) | -3.453 | 0.001 | 2659 | (0.114,0.414) | 0.36 |
| Distractibility | 3.95(0.57) | 3.97(0.70) | 0.277 | 0.782 | 2659 | (-0.154,0.122) | 0.03 |
| Threshold | 3.52(0.68) | 3.54(0.75) | 0.176 | 0.861 | 2659 | (-0.161,0.135） | 0.03 |
| EMBU |  |  |  |  |  |  |  |
| Emotional warmth | 2.69(0.59) | 2.70(0.54) | 0.194 | 0.847 | 95.928 | (-0.137,0.113) | 0.02 |
| Punishing | 1.89(0.63) | 1.55(0.54) | -5.782 | <0.001 | 2516 | (0.223,0.452) | 0.58 |
| Over-interference | 2.26(0.52) | 1.98(0.43) | -5.045 | <0.001 | 90.304 | (0.172,0.396) | 0.59 |
| Favoritism | 2.14(0.68) | 2.06(0.64) | -0.921 | 0.357 | 1747 | (-0.085,0.234) | 0.12 |
| Rejection | 1.85(0.61) | 1.56(0.52) | -5.564 | <0.001 | 2691 | (0.190,0.398) | 0.51 |
| Overprotection | 2.38(0.61) | 2.19(0.54) | -3.290 | 0.001 | 2649 | (0.075,0.297) | 0.33 |

UIs=unintentional injuries.

Because the outcome variable is a binary variable, binary logistic regression was used in the analysis.

| Variables | Binary logistic regression analysis | | | | |
| --- | --- | --- | --- | --- | --- |
|  | *P* | OR | *95% CI* | SE | *v* |
| Grade | <0.001 | 0.225 | （0.124，0.408） | 0.303 | 1 |
| Activity | 0.007 | 1.607 | （1.141，2.264） | 0.175 | 1 |
| Over-interference | 0.002 | 2.280 | （1.368，3.798） | 0.260 | 1 |

Confirmatory factor analysis.

TITLE: The structure of father

DATA: FILE IS C:\mplus\data\father.dat;

VARIABLE: NAMES ARE sex age u1-u4 f1-f5 t1-t10 ;

USEVARIABLES are t1 t2 t4 t6-t7 f2 f4;

ANALYSIS: ESTIMATOR=ML;

MODEL: s1 BY t1 t2 t4 t6-t7;

s2 by f2 f4;

t4 WITH t6;

OUTPUT: STANDARDIZED;

MODINDICES;

Mplus VERSION 6.12

MUTHEN & MUTHEN

06/07/2017 5:46 PM

INPUT INSTRUCTIONS

TITLE: The structure of father

DATA: FILE IS C:\mplus\data\father.dat;

VARIABLE: NAMES ARE sex age u1-u4 f1-f5 t1-t10 ;

USEVARIABLES are t1 t2 t4 t6-t7 f2 f4;

ANALYSIS: ESTIMATOR=ML;

MODEL: s1 BY t1 t2 t4 t6-t7;

s2 by f2 f4;

t4 WITH t6;

OUTPUT: STANDARDIZED;

MODINDICES;

INPUT READING TERMINATED NORMALLY

The structure of father

SUMMARY OF ANALYSIS

Number of groups 1

Number of observations 2614

Number of dependent variables 7

Number of independent variables 0

Number of continuous latent variables 2

Observed dependent variables

Continuous

T1 T2 T4 T6 T7 F2

F4

Continuous latent variables

S1 S2

Estimator ML

Information matrix OBSERVED

Maximum number of iterations 1000

Convergence criterion 0.500D-04

Maximum number of steepest descent iterations 20

Input data file(s)

C:\mplus\data\father.dat

Input data format FREE

THE MODEL ESTIMATION TERMINATED NORMALLY

MODEL FIT INFORMATION

Number of Free Parameters 23

Loglikelihood

H0 Value -13398.467

H1 Value -13361.950

Information Criteria

Akaike (AIC) 26842.934

Bayesian (BIC) 26977.913

Sample-Size Adjusted BIC 26904.835

(n* = (n + 2) / 24)

Chi-Square Test of Model Fit

Value 73.035

Degrees of Freedom 12

P-Value 0.0000

RMSEA (Root Mean Square Error Of Approximation)

Estimate 0.044

90 Percent C.I. 0.035 0.054

Probability RMSEA <= .05 0.827

CFI/TLI

CFI 0.992

TLI 0.986

Chi-Square Test of Model Fit for the Baseline Model

Value 7586.326

Degrees of Freedom 21

P-Value 0.0000

SRMR (Standardized Root Mean Square Residual)

Value 0.015

MODEL RESULTS

Two-Tailed

Estimate S.E. Est./S.E. P-Value

S1 BY

T1 1.000 0.000 999.000 999.000

T2 0.800 0.024 33.334 0.000

T4 0.849 0.028 29.834 0.000

T6 0.844 0.027 31.678 0.000

T7 1.289 0.034 37.721 0.000

S2 BY

F2 1.000 0.000 999.000 999.000

F4 0.889 0.068 13.050 0.000

S2 WITH

S1 0.060 0.006 10.081 0.000

T4 WITH

T6 0.054 0.005 10.384 0.000

Intercepts

T1 2.902 0.014 205.159 0.000

T2 3.042 0.011 275.870 0.000

T4 2.855 0.013 223.269 0.000

T6 2.857 0.012 240.601 0.000

T7 2.723 0.015 184.626 0.000

F2 1.565 0.010 149.979 0.000

F4 1.566 0.010 151.561 0.000

Variances

S1 0.257 0.013 19.301 0.000

S2 0.226 0.019 12.149 0.000

Residual Variances

T1 0.266 0.009 30.365 0.000

T2 0.153 0.005 29.162 0.000

T4 0.242 0.008 31.247 0.000

T6 0.185 0.006 30.006 0.000

T7 0.141 0.008 18.291 0.000

F2 0.059 0.017 3.442 0.001

F4 0.100 0.014 7.335 0.000

STANDARDIZED MODEL RESULTS

STDYX Standardization

Two-Tailed

Estimate S.E. Est./S.E. P-Value

S1 BY

T1 0.702 0.012 59.182 0.000

T2 0.720 0.012 62.034 0.000

T4 0.659 0.013 50.509 0.000

T6 0.705 0.012 59.457 0.000

T7 0.868 0.008 104.790 0.000

S2 BY

F2 0.891 0.034 26.502 0.000

F4 0.800 0.031 25.989 0.000

S2 WITH

S1 0.248 0.022 11.339 0.000

T4 WITH

T6 0.257 0.021 12.151 0.000

Intercepts

T1 4.013 0.059 68.194 0.000

T2 5.396 0.077 69.942 0.000

T4 4.367 0.063 68.787 0.000

T6 4.706 0.068 69.246 0.000

T7 3.611 0.054 67.326 0.000

F2 2.933 0.045 65.131 0.000

F4 2.964 0.045 65.259 0.000

Variances

S1 1.000 0.000 999.000 999.000

S2 1.000 0.000 999.000 999.000

Residual Variances

T1 0.508 0.017 30.534 0.000

T2 0.482 0.017 28.820 0.000

T4 0.566 0.017 32.905 0.000

T6 0.503 0.017 30.064 0.000

T7 0.247 0.014 17.212 0.000

F2 0.206 0.060 3.433 0.001

F4 0.359 0.049 7.293 0.000

STDY Standardization

Two-Tailed

Estimate S.E. Est./S.E. P-Value

S1 BY

T1 0.702 0.012 59.182 0.000

T2 0.720 0.012 62.034 0.000

T4 0.659 0.013 50.509 0.000

T6 0.705 0.012 59.457 0.000

T7 0.868 0.008 104.790 0.000

S2 BY

F2 0.891 0.034 26.502 0.000

F4 0.800 0.031 25.989 0.000

S2 WITH

S1 0.248 0.022 11.339 0.000

T4 WITH

T6 0.257 0.021 12.151 0.000

Intercepts

T1 4.013 0.059 68.194 0.000

T2 5.396 0.077 69.942 0.000

T4 4.367 0.063 68.787 0.000

T6 4.706 0.068 69.246 0.000

T7 3.611 0.054 67.326 0.000

F2 2.933 0.045 65.131 0.000

F4 2.964 0.045 65.259 0.000

Variances

S1 1.000 0.000 999.000 999.000

S2 1.000 0.000 999.000 999.000

Residual Variances

T1 0.508 0.017 30.534 0.000

T2 0.482 0.017 28.820 0.000

T4 0.566 0.017 32.905 0.000

T6 0.503 0.017 30.064 0.000

T7 0.247 0.014 17.212 0.000

F2 0.206 0.060 3.433 0.001

F4 0.359 0.049 7.293 0.000

STD Standardization

Two-Tailed

Estimate S.E. Est./S.E. P-Value

S1 BY

T1 0.507 0.013 38.602 0.000

T2 0.406 0.010 39.672 0.000

T4 0.431 0.012 35.362 0.000

T6 0.428 0.011 38.734 0.000

T7 0.654 0.013 51.507 0.000

S2 BY

F2 0.475 0.020 24.298 0.000

F4 0.423 0.018 23.510 0.000

S2 WITH

S1 0.248 0.022 11.339 0.000

T4 WITH

T6 0.054 0.005 10.384 0.000

Intercepts

T1 2.902 0.014 205.159 0.000

T2 3.042 0.011 275.870 0.000

T4 2.855 0.013 223.269 0.000

T6 2.857 0.012 240.601 0.000

T7 2.723 0.015 184.626 0.000

F2 1.565 0.010 149.979 0.000

F4 1.566 0.010 151.561 0.000

Variances

S1 1.000 0.000 999.000 999.000

S2 1.000 0.000 999.000 999.000

Residual Variances

T1 0.266 0.009 30.365 0.000

T2 0.153 0.005 29.162 0.000

T4 0.242 0.008 31.247 0.000

T6 0.185 0.006 30.006 0.000

T7 0.141 0.008 18.291 0.000

F2 0.059 0.017 3.442 0.001

F4 0.100 0.014 7.335 0.000

R-SQUARE

Observed Two-Tailed

Variable Estimate S.E. Est./S.E. P-Value

T1 0.492 0.017 29.591 0.000

T2 0.518 0.017 31.017 0.000

T4 0.434 0.017 25.254 0.000

T6 0.497 0.017 29.728 0.000

T7 0.753 0.014 52.395 0.000

F2 0.794 0.060 13.251 0.000

F4 0.641 0.049 12.995 0.000

QUALITY OF NUMERICAL RESULTS

Condition Number for the Information Matrix 0.178E-02

(ratio of smallest to largest eigenvalue)

MODEL MODIFICATION INDICES

NOTE: Modification indices for direct effects of observed dependent variables

regressed on covariates may not be included. To include these, request

MODINDICES (ALL).

Minimum M.I. value for printing the modification index 10.000

M.I. E.P.C. Std E.P.C. StdYX E.P.C.

WITH Statements

T2 WITH T1 33.124 0.030 0.030 0.149

T6 WITH T1 25.841 -0.026 -0.026 -0.118

T7 WITH T2 45.221 -0.043 -0.043 -0.293

Beginning Time: 17:46:49

Ending Time: 17:46:49

Elapsed Time: 00:00:00

Mediating effect analysis

TITLE: The structure of father SEM

DATA: FILE IS C:\mplus\data\father.dat;

VARIABLE: NAMES ARE sex age u1-u4 f1-f5 t1-t10 ;

USEVARIABLES are u4 f2 f4 t1 t2 t4 t6 t7 ;

CATEGORICAL = u4;

ANALYSIS: Bootstrap=1000;

MODEL: s1 BY f2 f4;

s2 by t1 t2 t4 t6 t7;

t6 WITH t4;

u4 on s2 s1 ;

s2 on s1;

MODEL INDIRECT:

u4 IND s2 s1;

OUTPUT: STANDARDIZED CINTERVAL(BCBOOTSTRAP);

Mplus VERSION 6.12

MUTHEN & MUTHEN

06/07/2017 5:55 PM

INPUT INSTRUCTIONS

TITLE: The structure of father SEM

DATA: FILE IS C:\mplus\data\father.dat;

VARIABLE: NAMES ARE sex age u1-u4 f1-f5 t1-t10 ;

USEVARIABLES are u4 f2 f4 t1 t2 t4 t6 t7 ;

CATEGORICAL = u4;

ANALYSIS: Bootstrap=1000;

MODEL: s1 BY f2 f4;

s2 by t1 t2 t4 t6 t7;

t6 WITH t4;

u4 on s2 s1 ;

s2 on s1;

MODEL INDIRECT:

u4 IND s2 s1;

OUTPUT: STANDARDIZED CINTERVAL(BCBOOTSTRAP);

INPUT READING TERMINATED NORMALLY

The structure of father SEM

SUMMARY OF ANALYSIS

Number of groups 1

Number of observations 2614

Number of dependent variables 8

Number of independent variables 0

Number of continuous latent variables 2

Observed dependent variables

Continuous

F2 F4 T1 T2 T4 T6

T7

Binary and ordered categorical (ordinal)

U4

Continuous latent variables

S1 S2

Estimator WLSMV

Maximum number of iterations 1000

Convergence criterion 0.500D-04

Maximum number of steepest descent iterations 20

Number of bootstrap draws

Requested 1000

Completed 1000

Parameterization DELTA

Input data file(s)

C:\mplus\data\father.dat

Input data format FREE

UNIVARIATE PROPORTIONS AND COUNTS FOR CATEGORICAL VARIABLES

U4

Category 1 0.962 2515.000

Category 2 0.038 99.000

THE MODEL ESTIMATION TERMINATED NORMALLY

MODEL FIT INFORMATION

Number of Free Parameters 26

WRMR (Weighted Root Mean Square Residual)

Value 0.660

MODEL RESULTS

Two-Tailed

Estimate S.E. Est./S.E. P-Value

S1 BY

F2 1.000 0.000 999.000 999.000

F4 0.888 0.063 14.001 0.000

S2 BY

T1 1.000 0.000 999.000 999.000

T2 0.830 0.025 32.757 0.000

T4 0.855 0.032 27.033 0.000

T6 0.849 0.031 27.819 0.000

T7 1.310 0.035 37.363 0.000

S2 ON

S1 0.269 0.030 8.901 0.000

U4 ON

S2 0.214 0.098 2.177 0.029

S1 0.493 0.083 5.936 0.000

T6 WITH

T4 0.058 0.006 9.617 0.000

Intercepts

F2 1.565 0.010 151.562 0.000

F4 1.566 0.010 153.137 0.000

T1 2.902 0.015 199.297 0.000

T2 3.042 0.011 283.229 0.000

T4 2.855 0.013 224.927 0.000

T6 2.857 0.012 240.267 0.000

T7 2.723 0.015 178.108 0.000

Thresholds

U4$1 1.776 0.045 39.572 0.000

Variances

S1 0.226 0.019 11.741 0.000

Residual Variances

F2 0.058 0.017 3.501 0.000

F4 0.101 0.013 7.556 0.000

T1 0.274 0.011 25.877 0.000

T2 0.146 0.006 24.469 0.000

T4 0.245 0.009 28.628 0.000

T6 0.189 0.007 26.315 0.000

T7 0.141 0.009 16.093 0.000

S2 0.233 0.012 18.848 0.000

STANDARDIZED MODEL RESULTS

StdYX StdY Std

Estimate Estimate Estimate

S1 BY

F2 0.892 0.892 0.476

F4 0.800 0.800 0.423

S2 BY

T1 0.690 0.690 0.499

T2 0.735 0.735 0.414

T4 0.653 0.653 0.427

T6 0.698 0.698 0.424

T7 0.867 0.867 0.654

S2 ON

S1 0.256 0.256 0.256

U4 ON

S2 0.107 0.107 0.107

S1 0.234 0.234 0.234

T6 WITH

T4 0.270 0.270 0.058

Intercepts

F2 2.933 2.933 1.565

F4 2.964 2.964 1.566

T1 4.013 4.013 2.902

T2 5.396 5.396 3.042

T4 4.367 4.367 2.855

T6 4.706 4.706 2.857

T7 3.611 3.611 2.723

Thresholds

U4$1 1.776 1.776 1.776

Variances

S1 1.000 1.000 1.000

Residual Variances

F2 0.204 0.204 0.058

F4 0.361 0.361 0.101

T1 0.524 0.524 0.274

T2 0.460 0.460 0.146

T4 0.574 0.574 0.245

T6 0.513 0.513 0.189

T7 0.249 0.249 0.141

S2 0.934 0.934 0.934

R-SQUARE

Observed Residual

Variable Estimate Variance

U4 0.079 0.921

F2 0.796

F4 0.639

T1 0.476

T2 0.540

T4 0.426

T6 0.487

T7 0.751

Latent

Variable Estimate

S2 0.066

TOTAL, TOTAL INDIRECT, SPECIFIC INDIRECT, AND DIRECT EFFECTS

Two-Tailed

Estimate S.E. Est./S.E. P-Value

Effects from S1 to U4

Sum of indirect 0.057 0.027 2.118 0.034

Specific indirect

U4

S2

S1 0.057 0.027 2.118 0.034

STANDARDIZED TOTAL, TOTAL INDIRECT, SPECIFIC INDIRECT, AND DIRECT EFFECTS

STDYX Standardization

Two-Tailed

Estimate S.E. Est./S.E. P-Value

Effects from S1 to U4

Sum of indirect 0.027 0.013 2.111 0.035

Specific indirect

U4

S2

S1 0.027 0.013 2.111 0.035

STDY Standardization

Two-Tailed

Estimate S.E. Est./S.E. P-Value

Effects from S1 to U4

Sum of indirect 0.027 0.013 2.118 0.034

Specific indirect

U4

S2

S1 0.027 0.013 2.118 0.034

STD Standardization

Two-Tailed

Estimate S.E. Est./S.E. P-Value

Effects from S1 to U4

Sum of indirect 0.027 0.013 2.111 0.035

Specific indirect

U4

S2

S1 0.027 0.013 2.111 0.035

CONFIDENCE INTERVALS OF MODEL RESULTS

Lower .5% Lower 2.5% Lower 5% Estimate Upper 5% Upper 2.5% Upper .5%

S1 BY

F2 1.000 1.000 1.000 1.000 1.000 1.000 1.000

F4 0.732 0.770 0.786 0.888 0.999 1.014 1.050

S2 BY

T1 1.000 1.000 1.000 1.000 1.000 1.000 1.000

T2 0.763 0.782 0.789 0.830 0.873 0.880 0.898

T4 0.765 0.787 0.798 0.855 0.904 0.912 0.931

T6 0.770 0.788 0.797 0.849 0.901 0.911 0.934

T7 1.223 1.245 1.259 1.310 1.374 1.385 1.402

S2 ON

S1 0.184 0.205 0.215 0.269 0.317 0.324 0.339

U4 ON

S2 -0.071 0.016 0.043 0.214 0.374 0.396 0.473

S1 0.270 0.314 0.354 0.493 0.618 0.644 0.706

T6 WITH

T4 0.043 0.047 0.049 0.058 0.069 0.071 0.074

Intercepts

F2 1.538 1.544 1.548 1.565 1.582 1.586 1.592

F4 1.542 1.547 1.550 1.566 1.584 1.587 1.593

T1 2.867 2.873 2.879 2.902 2.926 2.932 2.938

T2 3.014 3.022 3.025 3.042 3.060 3.064 3.071

T4 2.825 2.831 2.833 2.855 2.876 2.879 2.883

T6 2.827 2.835 2.838 2.857 2.878 2.881 2.887

T7 2.683 2.692 2.699 2.723 2.747 2.752 2.760

Thresholds

U4$1 1.659 1.686 1.702 1.776 1.850 1.861 1.895

Variances

S1 0.183 0.193 0.198 0.226 0.262 0.270 0.289

Residual Variances

F2 0.001 0.023 0.027 0.058 0.082 0.086 0.094

F4 0.067 0.076 0.081 0.101 0.124 0.130 0.139

T1 0.247 0.253 0.257 0.274 0.292 0.295 0.304

T2 0.132 0.135 0.138 0.146 0.157 0.159 0.164

T4 0.224 0.228 0.231 0.245 0.259 0.263 0.269

T6 0.172 0.176 0.178 0.189 0.203 0.206 0.209

T7 0.118 0.125 0.127 0.141 0.156 0.159 0.164

S2 0.204 0.209 0.212 0.233 0.253 0.259 0.265

CONFIDENCE INTERVALS OF TOTAL, TOTAL INDIRECT, SPECIFIC INDIRECT, AND DIRECT EFFECTS

Lower .5% Lower 2.5% Lower 5% Estimate Upper 5% Upper 2.5% Upper .5%

Effects from S1 to U4

Sum of indirect -0.017 0.005 0.012 0.057 0.102 0.110 0.129

Specific indirect

U4

S2

S1 -0.017 0.005 0.012 0.057 0.102 0.110 0.129

CONFIDENCE INTERVALS OF STANDARDIZED TOTAL, TOTAL INDIRECT, SPECIFIC INDIRECT,

AND DIRECT EFFECTS

STDYX Standardization

Lower .5% Lower 2.5% Lower 5% Estimate Upper 5% Upper 2.5% Upper .5%

Effects from S1 to U4

Sum of indirect -0.006 0.002 0.006 0.027 0.049 0.053 0.061

Specific indirect

U4

S2

S1 -0.006 0.002 0.006 0.027 0.049 0.053 0.061

STDY Standardization

Lower .5% Lower 2.5% Lower 5% Estimate Upper 5% Upper 2.5% Upper .5%

Effects from S1 to U4

Sum of indirect -0.006 0.002 0.006 0.027 0.049 0.053 0.061

Specific indirect

U4

S2

S1 -0.006 0.002 0.006 0.027 0.049 0.053 0.061

STD Standardization

Lower .5% Lower 2.5% Lower 5% Estimate Upper 5% Upper 2.5% Upper .5%

Effects from S1 to U4

Sum of indirect -0.006 0.002 0.006 0.027 0.049 0.053 0.061

Specific indirect

U4

S2

S1 -0.006 0.002 0.006 0.027 0.049 0.053 0.061

Beginning Time: 17:55:46

Ending Time: 17:58:58

Elapsed Time: 00:03:12

以父亲教养方式为中介

Take the father's parenting style as the intermediary

TITLE: The structure of father SEM

DATA: FILE IS C:\mplus\data\father.dat;

VARIABLE: NAMES ARE sex age u1-u4 f1-f5 t1-t10 ;

USEVARIABLES are u4 f2 f4 t1 t2 t4 t6 t7 ;

CATEGORICAL = u4;

ANALYSIS: Bootstrap=1000;

MODEL: s1 BY f2 f4;

s2 by t1 t2 t4 t6 t7;

t6 WITH t4;

u4 on s1 s2 ;

s1 on s2;

MODEL INDIRECT:

u4 IND s1 s2;

OUTPUT: STANDARDIZED CINTERVAL(BCBOOTSTRAP);

Mplus VERSION 6.12

MUTHEN & MUTHEN

06/08/2017 9:38 AM

INPUT INSTRUCTIONS

TITLE: The structure of father SEM

DATA: FILE IS C:\mplus\data\father.dat;

VARIABLE: NAMES ARE sex age u1-u4 f1-f5 t1-t10 ;

USEVARIABLES are u4 f2 f4 t1 t2 t4 t6 t7 ;

CATEGORICAL = u4;

ANALYSIS: Bootstrap=1000;

MODEL: s1 BY f2 f4;

s2 by t1 t2 t4 t6 t7;

t6 WITH t4;

u4 on s1 s2 ;

s1 on s2;

MODEL INDIRECT:

u4 IND s1 s2;

OUTPUT: STANDARDIZED CINTERVAL(BCBOOTSTRAP);

INPUT READING TERMINATED NORMALLY

The structure of father SEM

SUMMARY OF ANALYSIS

Number of groups 1

Number of observations 2614

Number of dependent variables 8

Number of independent variables 0

Number of continuous latent variables 2

Observed dependent variables

Continuous

F2 F4 T1 T2 T4 T6

T7

Binary and ordered categorical (ordinal)

U4

Continuous latent variables

S1 S2

Estimator WLSMV

Maximum number of iterations 1000

Convergence criterion 0.500D-04

Maximum number of steepest descent iterations 20

Number of bootstrap draws

Requested 1000

Completed 1000

Parameterization DELTA

Input data file(s)

C:\mplus\data\father.dat

Input data format FREE

UNIVARIATE PROPORTIONS AND COUNTS FOR CATEGORICAL VARIABLES

U4

Category 1 0.962 2515.000

Category 2 0.038 99.000

THE MODEL ESTIMATION TERMINATED NORMALLY

MODEL FIT INFORMATION

Number of Free Parameters 26

WRMR (Weighted Root Mean Square Residual)

Value 0.660

MODEL RESULTS

Two-Tailed

Estimate S.E. Est./S.E. P-Value

S1 BY

F2 1.000 0.000 999.000 999.000

F4 0.888 0.063 14.001 0.000

S2 BY

T1 1.000 0.000 999.000 999.000

T2 0.830 0.025 32.756 0.000

T4 0.855 0.032 27.032 0.000

T6 0.849 0.031 27.818 0.000

T7 1.310 0.035 37.361 0.000

S1 ON

S2 0.245 0.024 10.170 0.000

U4 ON

S1 0.493 0.083 5.936 0.000

S2 0.214 0.098 2.177 0.029

T6 WITH

T4 0.058 0.006 9.616 0.000

Intercepts

F2 1.565 0.010 151.564 0.000

F4 1.566 0.010 153.138 0.000

T1 2.902 0.015 199.297 0.000

T2 3.042 0.011 283.230 0.000

T4 2.855 0.013 224.930 0.000

T6 2.857 0.012 240.273 0.000

T7 2.723 0.015 178.107 0.000

Thresholds

U4$1 1.776 0.045 39.572 0.000

Variances

S2 0.249 0.013 19.071 0.000

Residual Variances

F2 0.058 0.017 3.501 0.000

F4 0.101 0.013 7.557 0.000

T1 0.274 0.011 25.876 0.000

T2 0.146 0.006 24.468 0.000

T4 0.245 0.009 28.628 0.000

T6 0.189 0.007 26.313 0.000

T7 0.141 0.009 16.091 0.000

S1 0.212 0.019 11.309 0.000

STANDARDIZED MODEL RESULTS

StdYX StdY Std

Estimate Estimate Estimate

S1 BY

F2 0.892 0.892 0.476

F4 0.800 0.800 0.422

S2 BY

T1 0.690 0.690 0.499

T2 0.735 0.735 0.414

T4 0.653 0.653 0.427

T6 0.698 0.698 0.424

T7 0.867 0.867 0.654

S1 ON

S2 0.256 0.256 0.256

U4 ON

S1 0.234 0.234 0.234

S2 0.107 0.107 0.107

T6 WITH

T4 0.270 0.270 0.058

Intercepts

F2 2.933 2.933 1.565

F4 2.964 2.964 1.566

T1 4.013 4.013 2.902

T2 5.396 5.396 3.042

T4 4.367 4.367 2.855

T6 4.706 4.706 2.857

T7 3.611 3.611 2.723

Thresholds

U4$1 1.776 1.776 1.776

Variances

S2 1.000 1.000 1.000

Residual Variances

F2 0.204 0.204 0.058

F4 0.361 0.361 0.101

T1 0.524 0.524 0.274

T2 0.460 0.460 0.146

T4 0.574 0.574 0.245

T6 0.513 0.513 0.189

T7 0.249 0.249 0.141

S1 0.934 0.934 0.934

R-SQUARE

Observed Residual

Variable Estimate Variance

U4 0.079 0.921

F2 0.796

F4 0.639

T1 0.476

T2 0.540

T4 0.426

T6 0.487

T7 0.751

Latent

Variable Estimate

S1 0.066

TOTAL, TOTAL INDIRECT, SPECIFIC INDIRECT, AND DIRECT EFFECTS

Two-Tailed

Estimate S.E. Est./S.E. P-Value

Effects from S2 to U4

Sum of indirect 0.120 0.022 5.413 0.000

Specific indirect

U4

S1

S2 0.120 0.022 5.413 0.000

STANDARDIZED TOTAL, TOTAL INDIRECT, SPECIFIC INDIRECT, AND DIRECT EFFECTS

STDYX Standardization

Two-Tailed

Estimate S.E. Est./S.E. P-Value

Effects from S2 to U4

Sum of indirect 0.060 0.011 5.390 0.000

Specific indirect

U4

S1

S2 0.060 0.011 5.390 0.000

STDY Standardization

Two-Tailed

Estimate S.E. Est./S.E. P-Value

Effects from S2 to U4

Sum of indirect 0.060 0.011 5.413 0.000

Specific indirect

U4

S1

S2 0.060 0.011 5.413 0.000

STD Standardization

Two-Tailed

Estimate S.E. Est./S.E. P-Value

Effects from S2 to U4

Sum of indirect 0.060 0.011 5.390 0.000

Specific indirect

U4

S1

S2 0.060 0.011 5.390 0.000

CONFIDENCE INTERVALS OF MODEL RESULTS

Lower .5% Lower 2.5% Lower 5% Estimate Upper 5% Upper 2.5% Upper .5%

S1 BY

F2 1.000 1.000 1.000 1.000 1.000 1.000 1.000

F4 0.732 0.770 0.786 0.888 0.999 1.014 1.050

S2 BY

T1 1.000 1.000 1.000 1.000 1.000 1.000 1.000

T2 0.763 0.780 0.789 0.830 0.873 0.879 0.898

T4 0.765 0.787 0.798 0.855 0.904 0.911 0.931

T6 0.770 0.788 0.797 0.849 0.901 0.911 0.934

T7 1.223 1.245 1.259 1.310 1.374 1.385 1.402

S1 ON

S2 0.184 0.192 0.201 0.245 0.281 0.288 0.304

U4 ON

S1 0.270 0.314 0.354 0.493 0.618 0.644 0.706

S2 -0.071 0.016 0.043 0.214 0.374 0.396 0.473

T6 WITH

T4 0.043 0.047 0.049 0.058 0.069 0.071 0.074

Intercepts

F2 1.538 1.544 1.548 1.565 1.582 1.586 1.592

F4 1.542 1.547 1.550 1.566 1.584 1.587 1.593

T1 2.867 2.873 2.879 2.902 2.926 2.932 2.938

T2 3.014 3.022 3.025 3.042 3.060 3.064 3.071

T4 2.825 2.831 2.833 2.855 2.876 2.879 2.883

T6 2.827 2.835 2.838 2.857 2.878 2.881 2.887

T7 2.683 2.692 2.699 2.723 2.747 2.752 2.760

Thresholds

U4$1 1.659 1.686 1.702 1.776 1.850 1.861 1.895

Variances

S2 0.220 0.224 0.228 0.249 0.271 0.276 0.286

Residual Variances

F2 0.001 0.023 0.027 0.058 0.082 0.086 0.094

F4 0.067 0.076 0.081 0.101 0.124 0.130 0.139

T1 0.247 0.253 0.257 0.274 0.292 0.295 0.304

T2 0.132 0.135 0.138 0.146 0.157 0.159 0.164

T4 0.224 0.228 0.231 0.245 0.259 0.263 0.269

T6 0.172 0.176 0.178 0.189 0.203 0.206 0.209

T7 0.118 0.125 0.127 0.141 0.156 0.159 0.164

S1 0.172 0.180 0.185 0.212 0.247 0.254 0.276

CONFIDENCE INTERVALS OF TOTAL, TOTAL INDIRECT, SPECIFIC INDIRECT, AND DIRECT EFFECTS

Lower .5% Lower 2.5% Lower 5% Estimate Upper 5% Upper 2.5% Upper .5%

Effects from S2 to U4

Sum of indirect 0.064 0.076 0.084 0.120 0.155 0.162 0.183

Specific indirect

U4

S1

S2 0.064 0.076 0.084 0.120 0.155 0.162 0.183

CONFIDENCE INTERVALS OF STANDARDIZED TOTAL, TOTAL INDIRECT, SPECIFIC INDIRECT,

AND DIRECT EFFECTS

STDYX Standardization

Lower .5% Lower 2.5% Lower 5% Estimate Upper 5% Upper 2.5% Upper .5%

Effects from S2 to U4

Sum of indirect 0.031 0.038 0.042 0.060 0.078 0.082 0.089

Specific indirect

U4

S1

S2 0.031 0.038 0.042 0.060 0.078 0.082 0.089

STDY Standardization

Lower .5% Lower 2.5% Lower 5% Estimate Upper 5% Upper 2.5% Upper .5%

Effects from S2 to U4

Sum of indirect 0.032 0.038 0.042 0.060 0.078 0.082 0.089

Specific indirect

U4

S1

S2 0.032 0.038 0.042 0.060 0.078 0.082 0.089

STD Standardization

Lower .5% Lower 2.5% Lower 5% Estimate Upper 5% Upper 2.5% Upper .5%

Effects from S2 to U4

Sum of indirect 0.031 0.038 0.042 0.060 0.078 0.082 0.089

Specific indirect

U4

S1

S2 0.031 0.038 0.042 0.060 0.078 0.082 0.089

Beginning Time: 09:38:36

Ending Time: 09:42:30

Elapsed Time: 00:03:54

MUTHEN & MUTHEN

3463 Stoner Ave.

Los Angeles, CA 90066

Tel: (310) 391-9971

Fax: (310) 391-8971

Web: www.StatModel.com

Support: Support@StatModel.com

Copyright (c) 1998-2011 Muthen & Muthen

父亲教养方式的中介：气质对非故意伤害发生的影响

Mediating paternal parenting style: The influence of temperament on the occurrence of unintentional injury

Mplus VERSION 6.12

MUTHEN & MUTHEN

01/09/2018 12:36 PM

INPUT INSTRUCTIONS

TITLE: The structure of father SEM

DATA: FILE IS C:\mplus\data\father.dat;

VARIABLE: NAMES ARE sex age u1-u4 f1-f5 t1-t10 ;

USEVARIABLES are u1 f2 f4 t1 t2 t4 t6 t7 ;

CATEGORICAL = u1;

ANALYSIS: Bootstrap=1000;

MODEL: s1 BY f2 f4;

s2 by t1 t2 t4 t6 t7;

t6 WITH t4;

u1 on s1 s2 ;

s1 on s2;

MODEL INDIRECT:

u1 IND s1 s2;

OUTPUT: STANDARDIZED CINTERVAL(BCBOOTSTRAP);

INPUT READING TERMINATED NORMALLY

The structure of father SEM

SUMMARY OF ANALYSIS

Number of groups 1

Number of observations 2614

Number of dependent variables 8

Number of independent variables 0

Number of continuous latent variables 2

Observed dependent variables

Continuous

F2 F4 T1 T2 T4 T6

T7

Binary and ordered categorical (ordinal)

U1

Continuous latent variables

S1 S2

Estimator WLSMV

Maximum number of iterations 1000

Convergence criterion 0.500D-04

Maximum number of steepest descent iterations 20

Number of bootstrap draws

Requested 1000

Completed 1000

Parameterization DELTA

Input data file(s)

C:\mplus\data\father.dat

Input data format FREE

UNIVARIATE PROPORTIONS AND COUNTS FOR CATEGORICAL VARIABLES

U1

Category 1 0.773 2021.000

Category 2 0.227 593.000

THE MODEL ESTIMATION TERMINATED NORMALLY

MODEL FIT INFORMATION

Number of Free Parameters 26

WRMR (Weighted Root Mean Square Residual)

Value 0.682

MODEL RESULTS

Two-Tailed

Estimate S.E. Est./S.E. P-Value

S1 BY

F2 1.000 0.000 999.000 999.000

F4 0.904 0.061 14.743 0.000

S2 BY

T1 1.000 0.000 999.000 999.000

T2 0.828 0.025 32.806 0.000

T4 0.855 0.031 27.272 0.000

T6 0.848 0.030 27.799 0.000

T7 1.302 0.035 37.349 0.000

S1 ON

S2 0.242 0.024 10.081 0.000

U1 ON

S1 0.429 0.060 7.147 0.000

S2 0.223 0.059 3.743 0.000

T6 WITH

T4 0.057 0.006 9.515 0.000

Intercepts

F2 1.565 0.010 151.569 0.000

F4 1.566 0.010 153.140 0.000

T1 2.902 0.015 199.286 0.000

T2 3.042 0.011 283.226 0.000

T4 2.855 0.013 224.923 0.000

T6 2.857 0.012 240.265 0.000

T7 2.723 0.015 178.103 0.000

Thresholds

U1$1 0.749 0.028 27.116 0.000

Variances

S2 0.250 0.013 19.177 0.000

Residual Variances

F2 0.062 0.015 4.062 0.000

F4 0.097 0.013 7.568 0.000

T1 0.273 0.011 25.767 0.000

T2 0.146 0.006 24.355 0.000

T4 0.244 0.009 28.551 0.000

T6 0.189 0.007 26.176 0.000

T7 0.144 0.009 16.216 0.000

S1 0.208 0.018 11.866 0.000

STANDARDIZED MODEL RESULTS

StdYX StdY Std

Estimate Estimate Estimate

S1 BY

F2 0.884 0.884 0.472

F4 0.807 0.807 0.426

S2 BY

T1 0.692 0.692 0.500

T2 0.734 0.734 0.414

T4 0.654 0.654 0.428

T6 0.698 0.698 0.424

T7 0.864 0.864 0.651

S1 ON

S2 0.257 0.257 0.257

U1 ON

S1 0.202 0.202 0.202

S2 0.111 0.111 0.111

T6 WITH

T4 0.267 0.267 0.057

Intercepts

F2 2.933 2.933 1.565

F4 2.964 2.964 1.566

T1 4.013 4.013 2.902

T2 5.396 5.396 3.042

T4 4.367 4.367 2.855

T6 4.706 4.706 2.857

T7 3.611 3.611 2.723

Thresholds

U1$1 0.749 0.749 0.749

Variances

S2 1.000 1.000 1.000

Residual Variances

F2 0.219 0.219 0.062

F4 0.349 0.349 0.097

T1 0.522 0.522 0.273

T2 0.461 0.461 0.146

T4 0.572 0.572 0.244

T6 0.512 0.512 0.189

T7 0.254 0.254 0.144

S1 0.934 0.934 0.934

R-SQUARE

Observed Residual

Variable Estimate Variance

U1 0.065 0.935

F2 0.781

F4 0.651

T1 0.478

T2 0.539

T4 0.428

T6 0.488

T7 0.746

Latent

Variable Estimate

S1 0.066

TOTAL, TOTAL INDIRECT, SPECIFIC INDIRECT, AND DIRECT EFFECTS

Two-Tailed

Estimate S.E. Est./S.E. P-Value

Effects from S2 to U1

Sum of indirect 0.104 0.017 6.012 0.000

Specific indirect

U1

S1

S2 0.104 0.017 6.012 0.000

STANDARDIZED TOTAL, TOTAL INDIRECT, SPECIFIC INDIRECT, AND DIRECT EFFECTS

STDYX Standardization

Two-Tailed

Estimate S.E. Est./S.E. P-Value

Effects from S2 to U1

Sum of indirect 0.052 0.009 6.085 0.000

Specific indirect

U1

S1

S2 0.052 0.009 6.085 0.000

STDY Standardization

Two-Tailed

Estimate S.E. Est./S.E. P-Value

Effects from S2 to U1

Sum of indirect 0.052 0.009 6.012 0.000

Specific indirect

U1

S1

S2 0.052 0.009 6.012 0.000

STD Standardization

Two-Tailed

Estimate S.E. Est./S.E. P-Value

Effects from S2 to U1

Sum of indirect 0.052 0.009 6.085 0.000

Specific indirect

U1

S1

S2 0.052 0.009 6.085 0.000

CONFIDENCE INTERVALS OF MODEL RESULTS

Lower .5% Lower 2.5% Lower 5% Estimate Upper 5% Upper 2.5% Upper .5%

S1 BY

F2 1.000 1.000 1.000 1.000 1.000 1.000 1.000

F4 0.744 0.784 0.802 0.904 1.002 1.023 1.058

S2 BY

T1 1.000 1.000 1.000 1.000 1.000 1.000 1.000

T2 0.761 0.778 0.786 0.828 0.869 0.877 0.896

T4 0.766 0.786 0.800 0.855 0.904 0.914 0.932

T6 0.768 0.788 0.798 0.848 0.901 0.909 0.930

T7 1.209 1.234 1.249 1.302 1.365 1.376 1.393

S1 ON

S2 0.180 0.190 0.200 0.242 0.279 0.287 0.302

U1 ON

S1 0.269 0.301 0.325 0.429 0.524 0.543 0.585

S2 0.069 0.111 0.131 0.223 0.325 0.342 0.389

T6 WITH

T4 0.043 0.046 0.048 0.057 0.068 0.070 0.073

Intercepts

F2 1.538 1.544 1.548 1.565 1.582 1.586 1.592

F4 1.542 1.547 1.550 1.566 1.584 1.587 1.593

T1 2.867 2.873 2.879 2.902 2.926 2.932 2.938

T2 3.014 3.022 3.025 3.042 3.060 3.064 3.071

T4 2.825 2.831 2.833 2.855 2.876 2.879 2.883

T6 2.827 2.835 2.838 2.857 2.878 2.881 2.887

T7 2.683 2.692 2.699 2.723 2.747 2.752 2.760

Thresholds

U1$1 0.684 0.694 0.704 0.749 0.794 0.801 0.818

Variances

S2 0.221 0.225 0.229 0.250 0.272 0.278 0.287

Residual Variances

F2 0.013 0.028 0.034 0.062 0.085 0.089 0.097

F4 0.065 0.073 0.078 0.097 0.119 0.123 0.134

T1 0.247 0.252 0.255 0.273 0.290 0.293 0.301

T2 0.132 0.136 0.138 0.146 0.158 0.159 0.165

T4 0.221 0.227 0.230 0.244 0.258 0.263 0.268

T6 0.172 0.176 0.178 0.189 0.203 0.206 0.209

T7 0.120 0.128 0.130 0.144 0.159 0.162 0.167

S1 0.171 0.179 0.184 0.208 0.242 0.251 0.266

CONFIDENCE INTERVALS OF TOTAL, TOTAL INDIRECT, SPECIFIC INDIRECT, AND DIRECT EFFECTS

Lower .5% Lower 2.5% Lower 5% Estimate Upper 5% Upper 2.5% Upper .5%

Effects from S2 to U1

Sum of indirect 0.063 0.074 0.078 0.104 0.133 0.139 0.153

Specific indirect

U1

S1

S2 0.063 0.074 0.078 0.104 0.133 0.139 0.153

CONFIDENCE INTERVALS OF STANDARDIZED TOTAL, TOTAL INDIRECT, SPECIFIC INDIRECT,

AND DIRECT EFFECTS

STDYX Standardization

Lower .5% Lower 2.5% Lower 5% Estimate Upper 5% Upper 2.5% Upper .5%

Effects from S2 to U1

Sum of indirect 0.030 0.035 0.038 0.052 0.066 0.069 0.074

Specific indirect

U1

S1

S2 0.030 0.035 0.038 0.052 0.066 0.069 0.074

STDY Standardization

Lower .5% Lower 2.5% Lower 5% Estimate Upper 5% Upper 2.5% Upper .5%

Effects from S2 to U1

Sum of indirect 0.030 0.035 0.038 0.052 0.066 0.069 0.074

Specific indirect

U1

S1

S2 0.030 0.035 0.038 0.052 0.066 0.069 0.074

STD Standardization

Lower .5% Lower 2.5% Lower 5% Estimate Upper 5% Upper 2.5% Upper .5%

Effects from S2 to U1

Sum of indirect 0.030 0.035 0.038 0.052 0.066 0.069 0.074

Specific indirect

U1

S1

S2 0.030 0.035 0.038 0.052 0.066 0.069 0.074

Beginning Time: 12:36:19

Ending Time: 12:36:57

Elapsed Time: 00:00:38

MUTHEN & MUTHEN

3463 Stoner Ave.

Los Angeles, CA 90066

Tel: (310) 391-9971

Fax: (310) 391-8971

Web: www.StatModel.com

Support: Support@StatModel.com

Copyright (c) 1998-2011 Muthen & Muthe
